# ‘Yebo, it was a great relief’: How mothers experience their children’s autism diagnoses

**DOI:** 10.4102/ajod.v12i0.1101

**Published:** 2023-03-28

**Authors:** Mbalenhle N. Manono, Mary G. Clasquin-Johnson

**Affiliations:** 1 Department of Inclusive Education, College of Education, University of South Africa, Pretoria, South Africa

**Keywords:** Afrocentrism, autism spectrum disorder, culture, diagnosis, maternal perspectives, mothers’ experiences, resilience, ubuntu

## Abstract

**Background:**

There is an emerging body of knowledge on the lived experiences of parenting a child with autism from a maternal perspective. Mothers’ reactions to their children’s autism diagnoses have been identified as a key factor influencing their children’s long-term outcomes.

**Objectives:**

This qualitative study aimed to explore how South African mothers experience their children’s autism diagnoses.

**Method:**

Telephonic interviews were conducted with 12 mothers from KwaZulu-Natal to understand their experiences prior, during and following their children’s autism diagnoses. The data were analysed thematically according to the values of *ubuntu*, social support, culture, tradition, interpersonal relationships, interconnectedness and continuity and compared to the existing scholarship, employing an Afrocentric theoretical lens.

**Results:**

The participants held strong cultural and religious beliefs which influenced the entire diagnosis process. Some, who waited a long time, turned to traditional healers or religious leaders. While some reported feeling relieved after the diagnosis, in the sense of at least having a name for their child’s condition, they also reported feeling overwhelmed by the realisation that there is no cure for autism. Over time, mothers’ feelings of guilt and anxiety declined, and they became increasingly resilient and empowered as their understanding of the meaning of their children’s autism diagnosis deepened, but many continued to pray for a miracle.

**Conclusion:**

Future research should focus on how to enhance support for mothers and their children during each of the three phases of autism diagnosis: prior, during and following their children’s autism diagnoses.

**Contribution:**

The study highlighted the crucial role of community-based religious and cultural organisations in providing appropriate support to mothers and their children diagnosed with autism, aligned to the values of *ubuntu*, social support, culture, tradition, interpersonal relationships, interconnectedness and continuity.

## Introduction

Autism spectrum disorder (ADS), or autism, is a complex, lifelong neurodevelopmental disorder characterised by difficulties related to social understanding and communication, repetitive or restricted behaviours and interests, as well as challenges related to adaptive functioning (American Psychiatric Association [APA] 2013). Zeidan et al. (2022) estimate that one out of one hundered children are diagnosed with ASD worldwide. Autism has been diagnosed in all races, ethnic and socio-economic groups and appears to be more prevalent in boys than girls (Kasari, Sturm & Shi 2018). The diagnosis of autism includes an evaluation of intellectual and language barriers to learning (Pillay & Brownlow 2017; Tonnsen et al. 2016). Scientists believe that both genetic and environmental factors are likely to play a role in autism (APA 2013; Sauer et al. 2021).

In this article, the authors illuminate the complex journeys of South African mothers whose children received an ASD diagnosis. While their experiences resemble maternal experiences globally, the authors seek to understand their lived experiences from an Afrocentric perspective, since existing scholarship confirms that early diagnosis combined with appropriate intervention leads to improved developmental and family outcomes (Farooq & Ahmed 2020). Mothers experience challenges caused by their child’s behaviour, influencing both mother and child’s acceptance within their communities (Shattnawi et al. 2021). Mothers’ challenges are associated with adverse psychosocial impacts because of their perceptions of loss, being judged by others, a lack of social support and personal distress (Wayment, Al-Kire & Brookshire 2019).

The family’s reactions to the child’s autism diagnosis have been identified as a major factor affecting long-term outcomes (Lord et al. 2018:3). Consequently, Lord et al. (2018) recommend that family members should be empowered with information related to resources and next steps when the autism diagnosis is communicated by healthcare professionals who are specially trained to do so.

Autism diagnosis frequently occurs after a lengthy, costly and stressful process (Clasquin-Johnson & Clasquin-Johnson 2018). Upon confirmation of an autism diagnosis, mothers may experience a range of emotions including shock, guilt and relief (DePape & Lindsay 2015; Lopez et al. 2018). Researchers characterise mothers’ experiences as a complex journey accompanied by mixed emotions (Crane et al. 2016; DePape & Lindsay 2015). While mothers are partly relieved to receive a diagnosis, they are also overwhelmed by the lifelong implications of autism (Lovelace, Robertson & Tamayo 2018). Over time, mothers’ feelings of guilt and anxiety decline, and they become increasingly resilient and empowered as their understanding of the meaning of their children’s autism diagnosis deepens (Marsh et al. 2017).

A study of 1000 mothers’ experiences of autism diagnoses in the United States of America revealed that mothers were more satisfied if: (1) there was a shorter time lag between initially seeking help and receiving a diagnosis, (2) diagnosis was received at a young age, (3) quality information on autism accompanied the diagnosis, (4) the professional who communicated the diagnosis framed autism in a positive manner and (5) they perceived intervention and support as accessible (Crane et al. 2016). Similarly, Webb et al. (2014) found that mothers tend to be more satisfied when their children are diagnosed during their early years. In addition, if mothers receive information on the nature of autism as well as how and where to access appropriate support, they are more satisfied with the diagnosis (Hoogsteen & Woodgate 2013; Webb et al. 2014). In contrast, if the process of obtaining a diagnosis is protracted, mothers may turn to alternative treatments such as dietary therapies and traditional healers, because of their reduced confidence in the healthcare professionals involved (Dougherty et al. 2016).

International studies have found that because of their caregiving burden, many mothers of children with autism experience high levels of psychological distress, health-related problems, lower levels of resilience and difficulties in family life, including marital and sibling relationships and family socialisation (Lovelace et al. 2018; Papadopoulos 2021). Some mothers experience chronic sadness and depression because of their children’s social and communication difficulties and challenging behaviour, which leads to self-accusation and negative attitudes towards life (Tekinarslan 2018).

Consequently, researchers have sought to identify protective factors for mothers of children diagnosed with autism (Muir & Strnadová 2014). Hope has been identified as a shielding or protective factor, as feeling hopeful reduces anxiety (Oprea & Stan 2012). In addition, when professionals establish a good rapport with mothers, their level of satisfaction increases, especially if mothers feel respected and included in decisions that benefit their children (Hoogsteen & Woodgate 2013).

In South Africa, there is a dearth of research on mothers’ experiences of the diagnosis of autism, although a few studies have focused on mothers’ experiences of their children’s diagnosis of a disability (Duma, Tshabalala & Mji 2021; Gow, Mostert & Dreyer 2020; Mkabile & Swartz 2020). It has also been noted that ‘There are no official statistics available on the prevalence of ASD among children in LMICs [low- and middle-income countries], including SA’ (Bakare & Munir 2011, cited in Erasmus et al. 2022:57). Consequently, most of the literature is from developed country contexts. This is the gap that this study aims to address. In this article, the authors will focus on mothers’ experiences prior, during and following their children’s diagnoses of autism.

### Theoretical approach

Asante (1983) defines Afrocentricity as ideas and actions shaped by African values, interests and perspectives. It promotes an appreciation for African identity and culture (Mkabela 2015; Richman 2018). The goal of Afrocentricity is to resituate African people ‘historically, economically, socially, politically, and philosophically’ (Asante 2003:3). This study was informed by Afrocentricity and the philosophy of *ubuntu*, which features concepts such as social support, culture, tradition, interpersonal relationships, spiritual interconnectedness and continuity. *Ubuntu* illuminates an African ethos, beliefs, experiences and aspirations (Bolden 2014; Majoko 2020; Mkabela 2015). These concepts should not be viewed as separate; rather, they are closely related, as they emphasise interconnectedness and reciprocity (Dolamo 2014; Gade 2015).

These concepts guided our data analysis and deepened our insights into mothers’ experiences from a communal and contextual perspective (Mkhize & Ndimande-Hlongwa 2014; Schiele, Gottschalk & Domschke 2020). According to African cultural values, anyone who experiences hardships should be treated with respect, receive support and be protected from marginalisation (Dolamo 2014). All children should benefit from shared wisdom and community networks of support, because the entire village (community) is responsible for raising all its children (Chaplin 2013; Gade 2015).

Human understanding is related to epistemology (ways of knowing) entrenched in culture (Baloyi 2015). In addition, African indigenous knowledge systems (IKS) formed a central element of this research (Asante 1987; Muwanga-Zake 2010). This compels the authors to question whether to approach autism from the dominant medical perspective of disability and a disease to be cured, or from a social inclusion perspective where the emphasis is on acceptance of diversity and the provision of support. The authors agree with the sentiments of Clasquin-Johnson (2020a:249), who explored African perspectives on autism, noting that:

In this era of decolonial and post-colonial discourse, how can we Africans allow the American Psychiatric Association to be the final arbiter of our (dis)ability? Is it not time for us to delve into the question of what it means to have a disability in African society? (p. 249)

The authors need to explore how connectedness, spirituality and traditional practices (Baloyi 2015) could play a supportive role for mothers and their children. In relation to this study, mothers may question what caused their child to have autism and may attribute autism to bewitchment or a reprimand for not following the expectations of their ancestors (Connolly & Gersch 2016; Mgbako & Glenn 2011). African mothers may seek counsel from an *iSangoma* (traditional healer) or *iNyanga* (herbalist) to understand autism (Madlala 2012), and those who seek to support them should respect these practices, viewing them as resources to enhance support.

African communities should facilitate a sense of belonging through the meaningful social inclusion of mothers and their children with autism. They can do this by eradicating stigma and discrimination associated with disability and difference (eds. Nsamenang & Tchombé 2012; Nussbaum 2013) and creating empowering conditions for children’s learning at home, at school and within their local communities (Murungi 2015). An Afrocentric perspective therefore holds promise for ‘seeing autism differently’ (Jansen 2020:iv) from a neurodiversity and differ-ability perspective (Clasquin-Johnson 2020b:17).

## Research methods and design

This exploratory study adopted a qualitative research approach (Choy 2014; Creswell 2014; McMillan & Schumacher 2014), to gain a better understanding of mothers’ experiences of their children’s diagnosis of autism. This enabled the authors to answer the main research question, ‘When children are diagnosed with autism, how do their mothers experience the process?’ The qualitative approach allowed for flexibility and increased participants’ freedom during the interviews (Maxwell & Saldaña 2014; Miles, Huberman & Saldaña 2014) to describe their experiences in as much detail as they chose. In addition, the semi-structured interviews were flexible and allowed the participants to share ‘complex and deep issues’ (Cohen, Manion & Morrison 2018:506) related to their experiences of their children’s autism diagnosis.

Twelve mothers participated in telephonic interviews, as this was most accessible and convenient for them, and in the context of the coronavirus disease 2019 (COVID-19) pandemic, this allowed the authors to adhere to the social distancing regulations in force at the time. The first author called the participants at arranged times, without requiring them to incur any costs.

### Study population and sampling strategy

The population of the study was mothers of children diagnosed with autism who were enrolled at a special school near Durban, KwaZulu-Natal. Purposive sampling was employed to obtain rich data related to mothers’ experiences of their children’s autism diagnoses in greater depth (ed. Given 2012). The mothers who granted informed consent and who volunteered to participate in the study were between the ages of 32 and 48 years, and their children were between the ages of 7 and 9. Prior to the interviews, each participant received a handwritten letter from the first author detailing the nature and purpose of the study. Table 1 outlines the profile of the twelve mothers who participated in this study.

**TABLE 1:** Profile of the participants.

Participant	Age of mother	Marital status	First language	Child’s present age	Child’s age of diagnosis	Child’s age at placement
Mother 1	43	Single	IsiZulu	8	5	7
Mother 2	38	Single	IsiZulu	8	5	7
Mother 3	45	Separated	IsiZulu	9	6	8
Mother 4	40	Single	IsiZulu	8	4	7
Mother 5	38	Married	IsiZulu	8	5	7
Mother 6	36	Divorced	IsiZulu	8	4	7
Mother 7	36	Divorced	IsiZulu	7	5	7
Mother 8	37	Single	IsiZulu	8	6	7
Mother 9	39	Single	IsiZulu	9	5	8
Mother 10	42	Separated	IsiZulu	8	5	8
Mother 11	33	Single	IsiZulu	7	5	6
Mother 12	32	Single	IsiZulu	7	4	6

*Source:* Manono, M.N., 2022, How mothers respond to autism diagnoses and navigate early intervention and support. Unpublished M Ed Dissertation. Pretoria: University of South Africa

With a single exception (Mother 5), all the participants were raising their children without the daily presence or involvement of their fathers. Seven mothers were identified as single, two as divorced, one as married and two as separated. The divorced and separated mothers noted that their marital relationships had ‘drifted apart’ following their children’s autism diagnoses. All the children spent an extended period on the special school’s waiting list before they were admitted.

### Data collection

During data collection, which was conducted in 2021, the authors complied with strict COVID-19 restrictions and protocols. Individual semistructured, in-depth interviews were conducted with the participants in their preferred language, either English or isiZulu, through telephone interviews that were audio-recorded and later transcribed verbatim and translated into English where necessary. The semistructured interviews allowed for an open, in-depth discussion on the 12 mothers’ experiences of the three phases of diagnosis. The first author put the mothers at ease and obtained their consent and trust before proceeding with the interviews, as recommended by Cohen et al. (2018). To ensure consistency, a semistructured interview scheduled was followed. The questions were developed according to the main research question and related research subquestions, aimed at gaining a deeper understanding of mothers’ experiences during each of the three phases of the diagnostic process.

### Data analysis

The authors relied on thematic analysis to analyse the data, focusing on recognising, evaluating and identifying patterns within the data according to the six steps proposed by Braun and Clarke (2021). To familiarise themselves with the data, the authors repeatedly read each participant’s transcribed and translated interview and assigned initial codes. This was followed by searching for possible themes, keeping the seven concepts related to Afrocentricity and the implications of IKS in mind. Thereafter, the authors carefully reviewed and refined the emerging themes and then defined and named them. Finally, the authors compiled a research report on the findings of the study. In addition, to ensure trustworthiness, the authors followed Denzin and Lincoln’s (2018) recommendations for credibility, dependability, transferability and confirmability of the study.

### Study context

The study was conducted at a special school for learners with a range of barriers to learning. At the time of data collection, there were 23 learners diagnosed with autism at the school. The mothers of 12 of the learners with autism participated in the study. It is worth noting that all 12 mothers were dependent on the public health system.

### Ethical considerations

Ethics clearance was sought and obtained from the College of Education’s Ethics Review Committee at the University of South Africa (ref. no. 2020/11/11/43638430/33/AM). The authors undertook to protect the participants from harm and ensure their privacy and confidentiality. The authors obtained permission from all 12 mothers to access documents such as their children’s learner profiles and medical records. These documents provided confirmation and detailed information on their diagnoses of autism as well as recommendations for school placement.

When inviting the 12 mothers to participate in the study, M.N.M. explained that there was a possibility that their participation in the study could cause distress related to sharing their past experiences of the process of their children’s diagnosis of autism (Rudestam & Newton 2015). The participants were advised of the availability of counselling by the school psychologist free of charge if the interview became too stressful (Ratcliff 2015). None of the participants took up this offer. Participants were further advised that they could withdraw from the study at any time without penalties. Throughout data collection, there was a great awareness of the need to remain respectful and compassionate when interacting with the 12 participants to advance their human rights and social justice, which the authors strive for in the field of inclusive education.

As discussed above, after the initial contact, all interviews were held telephonically to comply with the coronavirus disease 2019 (COVID-19) restrictions in place at the time.

## Results

Three research themes were generated from the data analysis performed by the authors: (1) mothers’ experiences prior to diagnosis, (2) mothers’ experiences during the diagnosis process and (3) mothers’ experiences following their children’s diagnoses. In the sections below, each theme and related subthemes will be discussed.

### Theme 1: Mothers’ experiences prior to diagnosis

Analysis revealed that all mothers had experiences prior to the diagnosis that influenced them to be intrinsically and/or extrinsically motivated to seek a diagnosis. At this stage (i.e. prior to the diagnosis), they were uncertain what the diagnosis would be. The following subthemes related to mothers’ experiences prior to their children’s autism diagnoses were: (1) intrinsic factors and (2) extrinsic factors. The intrinsic factors included psychological and emotional factors. The extrinsic factors included the mothers’ experiences of family and community members’ reactions to their children’s behaviour prior to diagnosis.

#### Intrinsic factors

Many of the mothers described feeling ‘confused’ and ‘frightened’ by their children’s early behavioural difficulties and developmental delays, as they struggled to reconcile it with their children’s typical appearance. For example, one mother described how her child ‘stared at objects endlessly’ and ‘spun around until he was dizzy’. Several mothers said that although they instinctively knew that something was different, their concerns were frequently dismissed by health professionals. Mothers’ instincts were a significant intrinsic factor that motivated them to pursue a diagnosis.

Mother 1 described her experiences as ‘completely chaotic and difficult’, as seen below:

‘It was completely chaotic because I was still dealing with being a first-time mother to a boy who was not developing at the normal pace. It was difficult, especially when you live far from hospitals and clinics in the location. While we were trying to get a diagnosis, we weren’t doing any therapies.’ (Mother 1, age 43, single)

Mother 7 described her instinctual awareness of her child’s problems.

‘There was just no language, but he cried a lot. He was short-tempered and he didn’t do what other kids do at his age. He was staring abnormally at one thing in the house. It looked like he was going to collapse, or he had lost his mind. I just knew … that something was not right with him.’ (Mother 7, age 36, divorced)

The extracts above reveal that the mothers experienced confusion, uncertainty and worry prior to their children’s autism diagnoses. Following the semistructured interview schedule, the mothers were guided to reflect on their emotions prior to their children’s autism diagnoses. They used the words, ‘disturbed’, ‘anxious’, ‘stressed’, ‘numb’, ‘frustrated’ and ‘hopeless’ to describe their emotions. Mother 3 articulated her confusion and uncertainty about her child’s developmental delays:

‘On the report was written “global development delay.” I took him to another hospital to attend speech therapy, physiotherapy, and all that. I received an appointment for three months later. I was not sure what was happening, just that he was delayed.’ (Mother 3, age 45, separated)

Mother 10 blamed herself for ‘overlooking the signs’, as revealed below:

‘I personally wish I never just sat hoping that he would get better and overlooking the signs before I had him assessed. I thought maybe it was in his father’s family … as his uncle is a stutterer.’ (Mother 10, age 42, separated)

These excerpts provide insight into the mothers’ emotions prior to their children’s diagnoses of autism. Mother 10 admitted that she initially ignored the warning signs and tried to find someone in the family with whom to associate her child’s behaviour. In the next section, mothers’ experiences of family and community members’ understanding of their children prior to diagnosis will be presented.

#### Extrinsic factors

As previously mentioned, all 12 mothers recognised that their children’s development was atypical or ‘delayed’. Mothers’ experiences included the reactions of family and community members to their children’s behaviour, which motivated them to seek a diagnosis. They sought advice from the elders in their families and communities. The feedback they received from the elders constituted a significant extrinsic factor that motivated them to seek a diagnosis. These reactions provide some insight into family and community members’ understanding of disability and difference.

Mother 2 shared that she felt ‘disturbed’ by her child’s father’s comments as well as her child’s atypical behaviour:

‘I was disturbed in my mind because his father likened him to a puppy because he was jumping on his toes, making funny noises, getting into people’s houses to steal food, and having no speech … He started making this high pitched “iiiiiiiii” sound.’ (Mother 2, age 38, single)

Mother 7 also described how she was influenced by the father’s remarks to delay seeking a diagnosis:

‘His father insisted that he was deaf, but I was not convinced until the audio screening. I took him to the hospital, nine months later. They said that they needed to do further tests because he failed the screening.’ (Mother 7, age 36, divorced)

Mothers 1, 3 and 6 discussed the lack of understanding in their respective religious communities:

‘We travelled by bus and would go to the hospital three times a week, but we weren’t seeing the correct people. My neighbour would take me to church where they did not understand my son.’ (Mother 1, age 43, single)

‘Some family members like his aunt and his uncle never understood what was going on. “*Ingane ayilashwe!*” [*They demanded that the child must be given proper herbs as he carries luck and truths for the family.*] They said that I must have caused him to be so.’ (Mother 3, age 45, separated)

‘My older brother told me that he is noticing something about him and that I should “*fanele kuyohlolw*a” [*consult a Sangoma*] because sometimes he groans like an animal. Maybe, “*ubiziwe*,” he has a calling to be a Sangoma and that has to be respected.’ (Mother 6, age 36, divorced)

These excerpts reveal the crucial role of family and community support. Mother 3 noted that when she turned to her family for support, they blamed her for her child’s difficulties since they believed that ‘I must have caused him to be so’, while also recognising that the child ‘carries luck and truths for the family’. Mother 6 was advised by her brother to ‘consult a Sangoma’, noting ‘that has to be respected’.

### Theme 2: Mothers’ experiences during the diagnosis process

When analysing mothers’ experiences during their children’s diagnoses of autism, two sub-themes emerged, namely: (1) diagnosis is a lengthy, stressful process, (2) relief to receive a diagnosis but worried about the future, because of the lack of guidance on intervention and support.

#### Diagnosis is a lengthy, stressful process

Several mothers indicated that they experienced most of the health care practitioners with whom they interacted during the diagnostic process as ‘unsupportive’ and ‘lacking in compassion’, which exacerbated their feelings of hopelessness and confusion. Some of the participants characterised their emotional distress as ‘extreme anxiety’ and ‘intense worry’, noting that the terminology carelessly used by the professionals intensified their confusion instead of providing clarity about autism and proving support. Some of the health care professionals speculated about the possible causes of the child’s behaviour without providing clear direction. This theme was especially noticeable in the following comments by Mothers 2, 6 and 7:

‘I was disturbed in my mind. For the whole first year, I was just numb. I did not even have the energy to go up and down for consultations with him. The word “autism” was all over his reports from the hospital. I would just stare at it and cry.’ (Mother 2, age 38, single)

‘I waited four months for an appointment. I took him to a so-called therapist, an occupational therapist. She checked him, and said he is fine. She said he can’t sit still and can’t concentrate, which I had also observed. She then said he might have ADHD [*attention deficit hyperactivity disorder*].’ (Mother 6, age 36, divorced)

‘It took us close to 8 months to get a consultation date with a paediatrician.’ (Mother 7, age 36, divorced)

The extracts above reveal the lengthy process of seeking a diagnosis.

Not only was diagnosis a lengthy process, but it was also experienced as stressful by the mothers. Key to this was the cold, impersonal attitude of medical professionals and the inflexible rules that must be followed:

‘I had to attend the specialist though she did not tell us what she was doing with the boy, and I had to help her to calm him down so that he could sit on the table. I don’t know English; neither does the child so we were not following *nje* [*laughing*].’ (Mother 6, age 36, divorced)

‘The doctor told me that he is hyperactive and so because he cannot concentrate, they will have to put him on medication, which I honestly wonder if it works because boy is the same. They gave me appointment dates far from each other. Even when the pills were messing him up, I could not just walk into the hospital, as I only had to go on my appointment dates.’ (Mother 2, age 38, single)

Indeed, mothers had to turn to unorthodox practices to get help:

‘You see at the hospital, now you will come with the child, maybe the medication has overturned his stomach, you must wake up at 4 am to be on the queue. Sometimes they tell you that the Risperdal is running out, come back three days later. You just have to know someone that knows someone inside in order to access help at times. You know how public hospitals are, *sisi*.’ (Mother 1, age 43, single)

‘There is a big public hospital in our zone behind the complex where we live. Well, I don’t have a problem saying it here, we live in South Africa ruled by the ruling party, so I would not go on the lines because of connections inside [*giggles*]. One nurse friend of mine organised for me to get his medication straight after bloods. Well, it’s the way it goes.’ (Mother 8, age 37, single)

#### Relieved to receive a diagnosis but worried about the future

The diagnosis was not accompanied by direction for their children, because the mothers were not provided with quality information on autism, nor was autism framed in a positive manner. Instead, they were more confused about the way forward for their children.

‘*Yebo,* it was a great relief. It was like, finally, we know the name of the disease and what it is called. Now I know what is abnormal about my son. What a breakthrough. But questions still remained whether it is curable or not.’ (Mother 1, age 43, single)

The mothers reported getting little guidance on what steps would follow diagnosis. Once again, they had to educate themselves by turning to the Internet, which was limited by the high cost of data.

‘I didn’t know what to do because nobody guided me on what the next step was and that was frustrating.’ (Mother 7, age 36, divorced)

### Theme 3: Mothers’ experiences following their children’s diagnoses

Based on the data analysis, two related subthemes were identified: (1) knowledge of autism and (2) personal adjustment. Each subtheme is discussed in more detail in the sections that follow.

#### Knowledge of autism

The mothers described how following their child’s diagnosis, they searched for the meaning of ‘autism’, constantly asking, ‘What is the Zulu name for this thing?’ and ‘Who has had it before?’ They turned to the elders in their families and communities and to traditional healers to undertake ‘*ukuyobhula’* or *‘ukuyozwa ababonayo*’ (those who can see through bones and make a diagnosis).

Again, the authors see an intrinsic–extrinsic division, where the mothers’ search for knowledge was intrinsic, while there was also the extrinsic influence of other people’s knowledge and acceptance.

For Mother 10, this stage actually preceded diagnosis and prompted her to seek formal acknowledgement of what she had discovered.

‘Someone mentioned Autism South Africa. I googled them even though I still didn’t comprehend what it was, so I didn’t worry much about it. Then after a while, as I read up on autism, I realised “*yabona*” [*you see*], it clarified some of the signs and then I decided to have my son assessed.’ (Mother 10, age 42, separated)

For the other participants, the information-seeking phase started after diagnosis:

‘Your heart is aching, and you wish to gather as much information as you can … I read all these books on parenting, and I watched a show on TV. The presenter said that when your child is tired, he won’t make eye contact. Besides it is only respectful to teach them to show respect by not making eye contact with the elders.’ (Mother 1, age 43, single)

‘I was committed to getting every single piece of information I possibly could on autism. Gosh, I just sat on the internet every moment after my son went to sleep. I was on my phone using the last money from his government grant. Until four or five in the morning, I was doing research on autism. But it was difficult to understand, and the data was expensive.’ (Mother 2, age 38, single)

‘My boy freaked me out. He would be like a robot, not talking, not smiling … At the time of the diagnosis, as this was my first child, I was lucky to come across Autism South Africa on the internet. I emailed them and described the challenges I was facing. As a young mother, I took advantage of technology and started reading on the search engines.’ (Mother 8, age 37, single)

These extracts reveal that following their children’s diagnoses, the mothers searched for the meaning of ‘autism’. Since quality information did not accompany the diagnosis, they had to find information on their own and were intrinsically motivated to do so. However, there were also extrinsic sources of information, remarks and behaviours of community members, which were largely informed by IKS.

‘*Yazi* [*you know what*], it was at the crèche where they did not accept my son because he kept messing in his trousers. I felt there was an implication that I didn’t do enough.’ (Mother 8, age 37, single)

‘In these past weeks my father, a traditional doctor himself, told me that I need to work on my parenting. See, if you ignore a lot of family issues, it manifests through the child. My father said that the elders in the family that have now passed, are living through this child and I should not force the child to speak our language as he is not of this world.’ (Mother 10, age 42, separated)

‘I was devastated, but the small section of my location [*township*] was already helping me to ensure the safety of my son because in a twinkling of an eye he would run into the road or even into another person’s house eating without permission. People accepted him though others took time to understand him.’ (Mother 11, age 33, single)

Autism awareness and acceptance were very limited in their families and communities. As seen above, they were blamed and criticised. Mother 8 noted, ‘I felt there was an implication that I didn’t do enough’, while Mother 10 noted her father’s comments:‘… I need to work on my parenting’. She added that ‘family issues’ ‘manifest through the child’. However, Mothers 10 and 11’s comments also suggest acceptance.

#### Personal adjustment

The mothers described their difficulties related to explaining their child’s diagnosis to others. In some cases, the mothers felt criticised that their children’s behaviour was attributed to their inadequate parenting skills. Over time, as support from close family members and friends (as well as their own understanding of autism) increased, they were less concerned about the opinions of others, especially strangers.

‘My colleagues at work and my sister understand my child’s condition. Other people in the street or at the supermarket don’t understand when he has a tantrum. They look at me very funny. And I know when he is throwing a tantrum that people will look at me.’ (Mother 7, age 36, divorced)

Religious and spiritual resources were employed by the mothers to supplement whatever medical and therapeutic interventions were available to them:

‘The mothers whom I pray with at the complex knew my son and their kids knew him too and that he is hyperactive. Also, other people started feeling empathy for me because they could see that I was stressed. Sometimes they would take him to the park to play with their kids because, *ya* I do not know how they knew that I was tired and needed rest and sleep, *nya* … We go to church every day. My faith is strong. Miracles do happen but I don’t feel so much pressure as my community members later started enquiring about autism and some would tell me that he is intelligent but can’t be still.’ (Mother 8, age 37, single)

‘I take him to Miracle Sundays. They told us to bring him consistently in the afternoons to claim his miracle. During Sunday School, he gains a lot because he socialises with other kids on the jungle gym. That is the only outing he ever takes with me [*breathing heavily*].’ (Mother 10, age 42, separated)

‘Church is the one place, I tell you, where we are greatly accepted. Social events like weddings, nah forget it. There [*at church*] he gets prayed for and I receive a lot of counselling for the entire week. It strengthens me.’ (Mother 11, age 33, single)

‘I decided yes, “*kuzohlatshwa*” [*goat slaughtering*] and the older men would speak and report that he is here and alive, that they should let go of him to be a normal child. We still do this to help him from time to time. He does say syllables now. Could I be winning?’ (Mother 2, age 38, single)

‘I won’t stop “*ukumhlabela*” [*meaning animal slaughter*] and put the wristband around his hand because you know four years ago, he did not say a sound but four years later as I try to sacrifice every year, he is starting to speak a language that nobody understands. He can point at water and say “*yuomeme*.” I think it’s the language known to the elders as they watch us.’ (Mother 3, age 45, separated)

## Discussion

In the context of the current study, autism diagnosis is a complex process that does not occur exclusively within the medical model of disability. The 12 participants held strong cultural and religious beliefs which influenced their experience of the process in all three phases, leading them to explore all avenues at their disposal, whether medical or traditional, and all forms of possible support. While Dougherty et al. (2016) contend that mothers who wait a long time for a diagnosis tend to lose confidence in the health care system and turn to traditional healers, this was not the case. Instead, the mothers *combined* the medical process with IKS and religion by seeking counsel from *iSangoma* or *iNyanga* (Madlala 2012) and/or Christian worship. Several mothers stated that their most urgent question was whether their children’s autism was attributable to bewitchment or their ancestors’ displeasure (cf. Connolly & Gersch 2016). The respondents did not necessarily refer to the concept of *ubuntu* directly; in fact, the data points to an impression that they experienced a lack of it. They found themselves isolated, without family, community or social acceptance, barring the occasional relative, colleague or friend. Instead, some were urged to find a boarding school since the community regarded autism as an illness to be cured and blamed the mother for the child’s challenges. Furthermore, the absence of coordinated collaboration among the different stakeholders increased mothers’ distress.

### Theme 1: Mothers’ experiences prior to diagnosis

The mothers were intrinsically and extrinsically motivated to seek a diagnosis because their children’s behaviour affected their acceptance within their communities (Shattnawi et al. 2021). This led to confusion and uncertainty prior to diagnosis (DePape & Lindsay 2015; Lopez et al. 2018; Lovelace et al. 2018). They turned to elders in their families for moral support and guidance when they noticed their children’s atypical behaviours and developmental delays. Although Mother 1 self-identified as a ‘first-time mother’, she recognised that her son was ‘not developing at the normal pace’. The mothers’ experiences were consistent with Teague et al.’s (2017) findings related to mothers’ fears of stigmatisation. These mothers were largely excluded from the benefits of collective wisdom and community support networks (Dolamo 2014). In many instances, those to whom they turned failed to provide social support by disregarding the interconnectedness of their reactions to the mothers’ hardships. This challenges the Afrocentric perspective and suggests that this model requires adjustment.

It is also significant that all but one of the 12 mothers were raising their children without the daily presence of their children’s fathers. Consistent with the findings of Lovelace et al. (2018) and Papadopoulos (2021), five of the mothers reported that their children’s autism diagnoses had adversely affected their marital relationships, which had ‘drifted apart’. Despite being absent, the children’s fathers’ negative comments appeared to increase the mothers’ anxiety, motivating them to seek a diagnosis. The mothers were responsible for executing the fathers’ advice; for example, Mother 1 recalled that her child’s father asked her to ‘do all the rituals required for his surname, so I took him to his father’s family for all that to be done’. The authors, therefore conclude that the lack of fathers’ involvement conflicts with the notion that ‘it takes a village to raise a child’, although they played a key role in reinforcing cultural beliefs and practices. The authors intend to explore these gender issues in a later publication.

### Theme 2: Mothers’ experiences during the diagnosis process

As noted by Weiss et al. (2016), professionals should provide appropriate support to reduce mothers’ anxiety and stress. Many of the health care professionals with whom the mothers interacted appeared to be unfamiliar with the characteristics of autism (as in the case of the therapist who initially suggested ADHD) and failed to prepare mothers for the possibility and implications of an autism diagnosis. The Zulu cultural emphasis on ‘*umuntu umuntu ngabantu*’ (meaning you are because others are), applies to the crucial role of health care professionals. While this is aligned to Afrocentrism, it is also firmly recommended in the international scholarship (Lord et al. 2018; Wayment et al. 2019). In these mothers’ experience, most health care professionals were unable to explain autism terminology (in particular, to give it a name in isiZulu) or provide information related to therapeutic and educational interventions as recommended by Crane et al. (2016) and Webb et al. (2014). The most direct expression of this was by Mother 7: ‘I didn’t know what to do because nobody guided me on what the next step was and that was frustrating’.

Several mothers pointed out that healthcare professionals lacked compassion because they could not relate to the mothers’ struggles. Their lack of empathy suggests that the health care professionals involved in diagnosis had not received adequate training on how to communicate a diagnosis as recommended by Hoogsteen and Woodgate (2013). Since many of the mothers were not treated with respect and compassion, nor were they provided with information that framed autism positively, they were dissatisfied with the process (Lord et al. 2018).

Consistent with Lovelace et al.’s (2018) findings, some mothers expressed relief to finally obtain a diagnosis after a frustrating, lengthy and stressful process because it gave a name for their child’s challenges, even though it failed to provide direction. It is also worth noting that all 12 mothers were dependent on the public health system, but the diagnosis process in the private health system is as lengthy because of a shortage of qualified personnel, and it is equally without direction (Clasquin-Johnson & Clasquin-Johnson 2018) but much more costly.

### Theme 3: Mothers’ experiences following their children’s diagnosis

Mothers’ reactions to their children’s autism diagnoses have been identified as a key factor influencing their children’s long-term outcomes (Lord et al. 2018). For this reason, mothers need to receive appropriate care and support, particularly since they are expected to be at the centre of their children’s intervention. As previously noted, prior to the children’s diagnoses, all but one of the mothers had limited prior knowledge of autism. They were unprepared for the shock of the diagnosis and experienced a general lack of autism awareness within their families, and communities. While four mothers received support from family members, neighbours and colleagues, this was uncommon. Several mothers described that animal sacrifices were frequently made within their families to heal both the child and the mother.

When seeking advice from individuals in key positions within their communities, the mothers needed to approach predominantly male *izindunas* (chiefs), *abakhuzis* (commanders), *izinyangas* and *ababonayos* (seers), since very few *sangomas* are women and mothers. It would therefore be crucial for these traditional leaders to understand mothers’ support needs, as this understanding would unlock (facilitate) *ubuntu*. Clearly, patriarchal power relations are a prominent barrier to acceptance. As stated above, the authors intend to explore this more deeply in an upcoming article.

When the mothers in this study sought religious support, the focus was placed on ‘healing’ rather than understanding their children’s challenges and support needs. At the African-initiated Christian churches to which most of the participants belonged, disability was viewed as a condition that required healing (cf. Amanze 2019). Despite this conflict between the religious and the medical positions, the churches became an important avenue of psychological and emotional support for the mothers. This is consistent with the recommendations of Muir and Strnadová (2014), as well as Oprea and Stan (2012), as it gave them hope for their children’s future.

All 12 mothers became increasingly resilient as they educated themselves on the meaning of their children’s autism diagnoses (cf. Marsh et al. 2017). Only one mother seemed to be aware of Autism South Africa, a national nongovernmental organisation that promotes autism awareness, and pose questions about autism to them via e-mail. Three mothers described how they conducted Google searches to learn more about autism. This is consistent with the literature that following the children’s diagnoses, the mothers were determined to learn as much as possible about autism (Kiami & Goodgold 2017). Consequently, all 12 mothers managed to enrol their children at an appropriate special school that accommodated children with autism.

### Implications

Although this study was conducted in KwaZulu-Natal and the participants were 12 Zulu mothers of children diagnosed with autism, it has raised awareness about the urgent need for culturally appropriate support for all persons diagnosed with ASD. Autism should be destigmatised through autism awareness programmes, targeting all stakeholders, including health, education, traditional, cultural and religious organisations, with useful information on the nature of autism, as well as how to access educational and therapeutic intervention and support within local communities (Hoogsteen & Woodgate 2013; Webb et al. 2014). This study reinforced the crucial role of community-based religious and cultural organisations in providing appropriate support to mothers and their children diagnosed with autism, aligned to the values of *ubuntu*, social support, culture, tradition, interpersonal relationships, interconnectedness and continuity (Majoko 2020; Mkabela 2015).

South Africa needs a systemic approach to autism diagnosis (Clasquin-Johnson & Clasquin-Johnson 2018), in which all the relevant stakeholders should be involved. This will ensure that mothers and their children derive the maximum benefit from autism interventions that respect religious and cultural diversity. In the short term, professionals at the frontline of the medical diagnosis process, as well as those at the frontline of cultural, traditional and religious support structures and institutions, require training on the characteristics of autism and the presentation of diagnosis, as they are vital in providing pre-, during- and post-diagnosis support to mothers and their children.
